# Smooth Extubation and Smooth Emergence Techniques: A Narrative Review

**DOI:** 10.1155/2021/8883257

**Published:** 2021-01-15

**Authors:** Tiffany H. Wong, Garret Weber, Apolonia E. Abramowicz

**Affiliations:** ^1^New York Medical College, Valhalla, NY, USA; ^2^Department of Anesthesiology, Westchester Medical Center, Valhalla, NY, USA

## Abstract

There is a paucity of literature on extubation technique and a lack of consensus regarding the definition of smooth extubation. This narrative review paper defines an ideal extubation, otherwise known as a “smooth extubation,” reviews perioperative criteria for extubation and risks and adverse events related to extubation, and explores various perioperative techniques that can be used to achieve a smooth extubation while caring for an uncomplicated patient without significant risk factors for extubation failure. In light of the evolving practice during the SARS CoV2 (COVID-19) pandemic to minimize aerosol generation and infection transmission, smooth extubation is particularly important.

## 1. Introduction

It has been recognized that tracheal extubation is associated with a significant risk of complications [1, 2]. Both the severity and frequency of extubation-related adverse events [3–6] and the paucity of literature regarding extubation techniques highlight a need to incorporate strategies for extubation in airway management guidelines [5, 7]. The Difficult Airway Society (DAS) developed a guideline for the management of tracheal extubation in 2012 [7]. This guideline focused on creating an extubation strategy before the onset of anesthesia, classified patients into low and high risk for extubation failure, and discussed general techniques on implementation of extubation [7].

While the DAS guideline provides an excellent starting point in developing strategies for achieving a successful extubation, it does not provide a distinction between successful extubation and smooth extubation. The concept of smooth emergence was mentioned in the DAS guideline as desirable for the success of certain surgical procedures, but it did not specify which procedures [7]. In addition, like much of the discussion regarding extubation techniques in the literature, a precise definition of “smooth extubation” was not stated [7].

It should be noted that the COVID-19 pandemic has heightened the importance of developing our knowledge of effective techniques to achieve smooth emergence. In an effort to reduce the transmission of COVID-19 to healthcare workers, various barriers or altered extubation setups have been devised to physically shield workers from aerosol and droplets generated during extubation [8–11]. Their effectiveness remains unknown, however. A smooth extubation may enhance primary prevention by reducing coughing, bucking, and aerosolization [12].

This narrative review will review the definition of smooth extubation and propose a specific definition for this term in an effort to suggest a uniform nomenclature facilitating interpretation and comparisons in future studies on smooth extubation. It will then discuss categories of adverse events associated with extubation in adults, although some of the referenced literature is based on a pediatric population. Finally, we will review different smooth extubation techniques, pharmacologic aids, and adjunct maneuvers to achieve smooth extubation success in the setting of perioperative care of an uncomplicated patient without significant risk factors for extubation failure. Although related, techniques for the smooth extubation in the difficult airway are beyond the scope of this review.

## 2. Definition of Smooth Extubation

As a result of limited practice guidelines and algorithms regarding extubation, there is a lack of a clear distinction on the difference between smooth and successful extubation. Successful extubation may indicate a purely respiratory level of success with patients tolerating the removal of the endotracheal tube, while a “smooth extubation” may include the lack of any physiologic responses that can lead to adverse outcomes from extubation. This distinction bears importance as many of the previous studies on criteria for successful extubation were conducted on critically ill patients on long-term ventilation [13]. The criteria for successful extubation in ICU patients may not apply to postoperative patients without significant risk for extubation failure.

Further, studies have mentioned “smooth extubation” and “smooth emergence” without a consensus of what these terms entail. We will use these terms interchangeably as they are often discussing the same desired outcome. The ambiguity on the factors of smooth emergence has led to the inclusion of different criteria in various papers. For example, a study by Lee et al. considered any coughing at all to be a failed smooth emergence [14]. Other studies expanded their definition of failed smooth extubation to include straining, movement, coughing, breath holding, or laryngospasm [15–17]. Hemodynamic perturbations, despite being potentially dangerous etiology of poor outcomes, were not considered as criteria in smooth emergence in either study.

We propose that smooth extubation should be a tranquil maneuver with minimal patient reaction and discomfort, stable vital signs, and maintenance of acceptable ventilation and oxygenation.

This definition may not be all-encompassing and further research is needed to elucidate usefulness of these criteria as a group in evaluating the success of smooth extubation and any additional factors that are necessary to ensure a smooth extubation.

## 3. Adverse Events Associated with Extubation

In the context of extubation, adverse events can be sorted into three major categories: respiratory, traumatic, and hemodynamic. Many of these adverse events can result in the depletion of oxygen stores at extubation, resulting in postoperative hypoxia. In severe cases, hypoxia can lead to hypoxic brain injury, cardiovascular injury, and possibly death [18]. Furthermore, it has been noted that a majority of emergency tracheal reintubations after perioperative extubation were due to preventable anesthesia-related factors [19]. These adverse events, summarized in Table 1, should be avoided with a smooth emergence.

### 3.1. Respiratory

Approximately 5% of patients undergoing inpatient noncardiac surgery experience a major pulmonary complication which is associated with significant risk of mortality [20, 21]. Many of the respiratory effects of extubation are due to the alteration of laryngeal reflexes. Exaggerated laryngeal reflexes can result in coughing, bucking, or laryngospasm. Coughing and bucking may increase arterial pressure, heart rate, and intraocular and intracranial pressure. Bucking is defined as a more forceful and prolonged cough which can physiologically imitate a Valsalva maneuver but at variable lung volumes, potentially leading to hypoxemia. Protracted bucking can also rarely lead to abdominal wound separation [13]. Reduced airway muscle responses, resulting in decreased airway tone and obstruction, are also associated with adverse outcomes. Laryngeal functional disturbance can lead to an inability to sense foreign material for at least four hours, even in apparently alert patients [22], increasing the risk of aspiration and pneumonia. Pulmonary aspiration of gastric contents was the third most common respiratory event leading to anesthesia malpractice claims from 1990–2007 [5]. In the Fourth National Audit Project of the Royal College of Anesthetists (NAP4), aspiration was found to be the most common primary cause of death in anesthesia-related events [3]. The most common reason for reduced airway patency and decreased reactivity postoperatively is thought to be the relaxation of pharyngolaryngeal muscles due to residual effects of inhalational anesthetics and/or the inadequate reversal of neuromuscular blockade, leading to significant risk of postoperative pulmonary complications [21, 23]. While neostigmine and glycopyrrolate have traditionally been used to reverse neuromuscular blockade in the United States, Sugammadex was introduced for reversal of neuromuscular blockade in 2015. [21] Recent studies have shown a 30% reduced risk of pulmonary complications, 47% reduced risk of pneumonia, a 55% reduced risk of respiratory failure, and reduction in odds of reintubation for respiratory failure or new noninvasive ventilation with the use of Sugammadex compared to neostigmine [21, 24]. This favors the use of Sugammadex for most extubations.

#### 3.1.1. Laryngospasm

In the context of extubation, laryngospasm is the most common cause of upper airway obstruction, occurring most often after removal of the endotracheal tube. Laryngospasm has previously been narrowly defined as an occlusion of the glottis by the reflexive action of the intrinsic laryngeal muscles. In addition to the glottis, closure of the larynx due to laryngospasm can also occur at all levels of the upper airway, including the aryepiglottic folds and the false vocal cords [25]. Laryngospasm is precipitated by local irritation of the vocal cords by stimuli such as secretions, blood, inflow of cold, dry air from oxygen flush from anesthesia circuit, or a foreign body including an endotracheal tube [13, 25]. It serves as a protective reflex to prevent foreign material from entering the tracheobronchial tree but can be detrimental in the perioperative setting as the closure of the airway in laryngospasm can continue beyond the cessation of the provoking stimulus. This can lead to complete airway obstruction with hypoxia and hypercarbia, and when untreated, can result in negative pressure pulmonary edema, and even hypoxic cardiac arrest [7].

#### 3.1.2. Negative Pressure Pulmonary Edema

Negative pressure pulmonary edema, also known as postobstructive pulmonary edema, is most commonly caused by laryngospasm but can occur as a result of any cause of complete airway obstruction, which can happen at the time of extubation. The pathogenesis is multifactorial, but the dominant mechanism is likely due to forceful inspiratory efforts against an obstructed airway causing negative intrathoracic pressure leading to pulmonary edema [2]. The treatment often requires endotracheal reintubation, mechanical ventilation, and diuresis [26].

### 3.2. Trauma

Difficult extubation can result in trauma to the upper and lower airways, most commonly resulting in damage to the larynx and the vocal cords. According to the ASA Closed Claims Database, 80% of the laryngeal nerve injuries followed routine airway management [4]. Although occurring at intubation, the injuries manifest at or after extubation. Excessive suctioning during extubation has also been noted to cause trauma to the mobile structures in the upper airway, such as the arytenoid cartilages [2]. Injury to the vagus nerve or the recurrent laryngeal nerve can cause vocal cord paralysis. Bilateral vocal cord paralysis can cause airway obstruction that can require emergent reintubation; this may rarely occur after head and neck surgery [2]. Local oropharyngeal trauma with extubation can result in edema, causing airway compromise requiring reintubation [2]. Anesthesia-related dental trauma has been noted to occur more commonly during intubation [27], but 9–20% of these injuries occur during extubation or in the recovery room [28]. Laryngeal injuries range in severity from minor, soft tissue edema, erythema, hematoma, ulceration, mass lesions, stenosis, and immobility of the vocal cords [29]. Lingual trauma, such as laceration due to a patient biting their tongue during emergence, or lingual hematoma, has also been documented, but the incidence during extubation is unknown. Various bite blocks are in use to prevent lingual trauma; nevertheless, severe pressure injury to the tongue has been reported which becomes apparent upon extubation [30]. Emotional distress can also result from awareness during extubation, potentially leading to deleterious sequelae such as symptoms of posttraumatic stress disorder and a permanent phobia to surgery and anesthesia [31–33]. Finally, as mentioned above, protracted bucking can lead to surgical incision breakdown as well as impaired ventilation leading to hypoxia [13].

### 3.3. Hemodynamic Shifts

The act of extubation itself is physiologically stressful and has been shown to cause increases in blood pressure and heart rate [13, 15, 34–36]. In addition, coughing can lead to increases in intrathoracic pressure which can interfere with venous return to the heart [37]. Drugs commonly used in preparation for extubation, such as glycopyrrolate, can cause tachycardia which leads to an increase in myocardial demand and can result in myocardial ischemia [38]. While transient hemodynamic shifts as a result of extubation are usually well tolerated in patients without coexisting disease, some patients demonstrate an exaggerated response [13]. Patients who have undergone neurosurgical operations are particularly sensitive to postoperative disturbances of autoregulation of cerebral blood flow, with hypertension resulting in brain hemorrhage and/or herniation [2].

## 4. Smooth Emergence Techniques

### 4.1. Deep Extubation

Although awake and deep extubation may have similar risks when incorrectly performed, anesthesiologists often associate deep extubation with an increased risk of aspiration, laryngospasm, and loss of airway control compared to awake extubation [39]. Despite its perceived risks, deep extubation can also help avoid many of the potential airway irritant and hemodynamic complications, such as coughing/bucking, tachycardia, and hemodynamic swings [39].

Several studies reported decreased incidence of persistent cough with deep extubation with no change in overall incidence of perioperative adverse events between deep extubation and awake extubation [40–42]. A recent systematic review and meta-analysis of deep extubation in a pediatric population found that while deep extubation reduces the risk of overall airway complications, there is an increased risk of airway obstruction. Further, there was no difference in the incidence of laryngospasm between the deep and awake extubation group [43].

Given the lack of data, anesthesiologists often rely on clinical experience when deciding to perform a deep extubation. In a study surveying extubation practice in adult surgical patients, the most common reasons cited for not performing deep extubation were lack of necessity and concern regarding potential laryngospasm and aspiration [37]. Based upon the available evidence, these concerns might not be fully supported and should be reassessed.

Furthermore, deep extubation can be combined with different techniques to increase the success rate of smooth emergence. A study comparing deep extubation with desflurane and remifentanil with a control of only desflurane showed a reduction in recovery time and incidence of respiratory complications in the combined desflurane/remifentanil group [44]. Another study investigated smooth emergence of adult patients with otologic surgery after deep extubation with inhaled anesthetic agent combined with either remifentanil (0.03 mcg/kg over 10-minute infusion), dexmedetomidine (0.5 mcg/kg over 10-minute infusion), or dexmedetomidine (0.7 mcg/kg over 10-minute infusion). While the outcomes were not compared to a control group of awake extubation, only one patient in the study developed desaturation and laryngospasm, and no patient required reintubation. All groups had low incidence of cough during extubation. The dexmedetomidine 0.7 mcg/kg and the remifentanil groups provided similar rates of smooth emergence compared to the dexmedetomidine 0.5 mcg/kg group. In addition, both dexmedetomidine groups had the added advantage of opioid sparing effects and less postoperative nausea and vomiting than remifentanil [45]. It is reasonable to infer from this study that dexmedetomidine may be an underutilized tool in clinical practice to achieve smooth extubation. The reasons for underutilization may include concerns regarding added cost [46] or delayed emergence [47].

### 4.2. ETT Exchange to Laryngeal Mask Airway (LMA)

LMAs have been shown to be a possibly useful adjunct in achieving smooth emergence [48]. In a study of 60 patients, patients were treated with either traditional awake removal of ETT or exchange of ETT with LMA followed by awake extubation with LMA. The incidence of both respiratory complications and significant hemodynamic shifts was decreased in the group with LMA exchange [49]. A similar study achieved the same results in elderly patients with hypertension [50].

### 4.3. “No-Touch” Extubation

A “no-touch” extubation is a technique in which absolutely no stimulation is allowed until patients spontaneously wake up. Tsui et al. reported successful “no-touch” extubation of twenty patients in whom there were no instances of laryngospasm or coughing [51]. Similarly, Sheta et al. noted a decrease in the incidence of laryngospasm in the “no-touch” group compared to the control group, awakened with stimuli. Furthermore, the “no-touch” extubation had significantly less severe cough, excess secretions, breath holding, hoarseness, biting, occurrence of nonpurposeful movements, and changes in heart rate, systolic blood pressure, and diastolic blood pressure. However, there was no significant difference in the incidence of postoperative sore throat [52].

## 5. Pharmacologic Aids of Tracheal Extubation

A variety of pharmacological treatments have been studied to improve emergence. The efficacy of these medications is often difficult to compare due to the use of different definitions of smooth extubation and studies measuring different effects. Many of these studies are often also subject to small sample sizes and are usually tested on ASA I-II patients undergoing elective surgeries. It should be noted that these pharmacologic aids may not have the same efficacy in high-risk patients. Despite these limiting factors, we discuss some of the more widely studied medications below (Table 2) [13, 15, 34–36, 45, 47, 53–56, 59–63]. Other pharmacologic agents, such as ketamine, have been used to achieve smooth emergence [64]. However, there is less published literature on its efficacy in the setting of smooth extubation with adults and it will not be discussed.

### 5.1. Intracuff Lidocaine

The cuff of the endotracheal tube (ETT) has been suggested as a potential reservoir for local anesthetics, such as lidocaine, to decrease the incidence of postoperative sore throat and cough. There have been some concerns that lidocaine would not be able to diffuse through the ETT cuff. However, in vitro and in vivo studies have shown that lidocaine is able to diffuse through the ETT cuff, albeit rather slowly [65, 66]. Alkalinization or a combination of warming and alkalinization of the anesthetic can increase the proportion of the uncharged drug available for diffusion, thereby increasing the rate of diffusion [67]. Many studies have demonstrated the efficacy of intracuff lidocaine in decreasing cough and sore throat [53–55]. Two systematic reviews and meta-analyses demonstrated that both alkalinized and nonalkalinized intracuff lidocaine showed significant reduction of postoperative sore throat, coughing, and dysphonia compared to control groups without intracuff lidocaine [53, 54].

### 5.2. Intravenous (IV) Lidocaine

IV lidocaine has long been studied as an agent to suppress laryngospasm and the cardiovascular response to extubation [13, 34, 35]. However, it should be noted that evidence from other studies has not always supported the efficacy of lidocaine to attenuate or prevent these changes [2]. A systematic review and meta-analysis of the use of IV lidocaine to achieve smooth extubation showed that treatment led to large reductions in postextubation cough and sore throat. However, there was no difference in the incidence of laryngospasm or adverse events compared to the control [56].

### 5.3. Topical Lidocaine, Methylprednisolone, and Benzydamine Hydrochloride

In addition to intracuff and IV delivery of lidocaine, lidocaine can also be administered topically in the oropharynx and glottis. Zamora Lozano et al. demonstrated that topical administration is not as effective as intracuff or IV administration in reducing the incidence of cough during emergence [55]. In addition, application of lidocaine spray to the oropharyngeal cavity before intubation seems to increase the incidence of postoperative sore throat in a dose-dependent manner [59]. Furthermore, Watkins et al. found that topical lidocaine increased the mean times for extubation by nearly 2 minutes [62]. In comparison, topical methylprednisolone [61] and ETT cuff coated benzydamine hydrochloride [59, 60] have both been shown to improve postoperative sore throat compared to topical lidocaine but are not as widely used in clinical practice.

### 5.4. IV Dexmedetomidine

Dexmedetomidine can be used as a sedative agent without the added risk of respiratory depression [63] and can aid in smooth emergence. Bindu et al. demonstrated that IV infusion of 0.75 microgram per kilogram (mcg/kg) of dexmedetomidine fifteen minutes before the end of surgery reduced the amount of coughing upon emergence [15]. Patients treated with dexmedetomidine were slightly more sedated upon emergence and had increased incidence of bradycardia and hypotension, but no significant sequelae as a result [15]. Fan et al. found that a 10-minute IV infusion of 0.5 mcg/kg or 0.7 mcg/kg of dexmedetomidine for deep extubation resulted in an increased rate of smooth emergence, increased respiratory rate, decreased need for rescue analgesic, and less postoperative nausea and vomiting compared to extubation with 10-minute infusion of 0.04 mcg/kg of IV remifentanil [45]. A study by Guler et al. found that there were decreased agitation and pain scores and decreased amount of severe coughing with treatment with 0.5 mg/kg IV dexmedetomidine 5 minutes before the end of surgery compared to placebo. However, times to emergence were significantly longer in the treatment group [47]. A systematic review and meta-analysis of 0.4 mcg-0.5 mcg/kg of dexmedetomidine supports its use to achieve smooth extubation without causing respiratory depression. However, higher doses resulted in bradycardia, hypotension, and sedation [68]. Dexmedetomidine will be discussed further below in combination with deep extubation.

### 5.5. IV Remifentanil

Remifentanil is an ultrashort acting opioid which can be used to achieve smooth emergence. A study by Nho et al. found that IV infusion of remifentanil via target controlled infusion system to target concentration of 1 nanogram per milliliter at the end of surgery attenuated increases in heart rate and incidence of moderate or severe coughing during emergence compared to the control group. The mean arterial pressure was also lower in the treatment group during recovery [36]. However, as discussed previously, remifentanil was not as effective as dexmedetomidine at facilitating smooth emergence [45]. It should be noted that other opioids, such as fentanyl and tramadol, may be used as an aid in smooth extubation [16, 69].

### 5.6. IV Calcium Channel Blockers

Calcium channel blockers, such as verapamil and diltiazem, can be used to attenuate the hemodynamic responses to emergence. A study by Mikawa et al. found that IV administration of verapamil 0.05 mg/kg, verapamil 0.1 mg/kg, or diltiazem 0.2 mg/kg two minutes before extubation could attenuate increases in heart rate as well as systolic and diastolic pressure compared to a control group of IV saline. 0.1 mg/kg of verapamil had the greatest effect on the attenuation of the hemodynamic changes. None of the patients developed hypotension, bradycardia, or sinoatrial or atrioventricular block severe enough to warrant intervention [58]. Another study by Swamy and Madhusudhana found that a combined dose of IV administration diltiazem 0.2 mg/kg or 0.1 mg/kg and lidocaine 1.0 mg/kg was more effective at attenuating hemodynamic changes than just 1.0 mg/kg lidocaine alone [57].

## 6. Adjunct Maneuvers to Smooth Extubation

In addition to pharmaceutical adjuncts, there are also several adjunct maneuvers that have been studied to improve emergence (Table 3).

### 6.1. Preoxygenation with 100% Fraction of Inspired Oxygen (FiO2) prior to Extubation

Preoxygenation with 100% FiO2 should be considered at emergence to maximize pulmonary oxygen stores. This allows for functional reserve capacity of oxygen available in case of airway obstruction or unanticipated hypoxia [13, 18, 48]. There have been some concerns that preoxygenation with 100% FiO2 prior to extubation can lead to worsened atelectasis in the postoperative setting [70, 71]. However, there is a lack of sufficient evidence that preextubation oxygenation causes postoperative hypoxia due to atelectasis or other clinically significant untoward effects on patients [72].

### 6.2. Bite Blocks

Upon emergence, patients may become agitated or delirious and bite down on the endotracheal tube. This has resulted in partial occlusion [84–88] or complete transection of the endotracheal tube [89], causing airway obstruction [84, 85, 88], and negative pressure pulmonary edema [90]. Thus, bite blocks should be used during emergence to prevent such an occurrence [7]. There is insufficient evidence to demonstrate whether soft bite blocks, such as rolled or taped gauze, or hard bite blocks are better at maintaining airway patency during extubation. There has been some support for use of soft bite blocks as opposed to hard bite blocks in preventing oral injuries, as hard bite blocks may cause pressure injuries and dental trauma [91]. An oropharyngeal airway has been suggested as a hard bite block which simultaneously provides a patent upper airway [7]. However, the oropharyngeal airway does not always prevent a patient from biting down and occluding an endotracheal tube [73] and its use can potentially lead to dental trauma [74], aspiration of the oropharyngeal airway [75], and dislodgement into the esophagus [76].

### 6.3. Patient Monitoring and Supplemental Oxygen Use during Transfer to Recovery Room

Hypoxemia can lead to severe adverse outcomes. Postoperative hypoxemia occurs commonly [74] and has been documented even after minor procedures [77] and may remain unrecognized unless the patient is monitored with a pulse oximeter [78]. Ventilatory depression likewise may go unrecognized, unless capnography is utilized [3]. Patients are particularly vulnerable to hypoxemia during transfer from the operating room to the recovery room as ventilation is likely to be depressed from residual anesthetics and other sedatives used in general anesthesia [79, 80]. Despite the frequency and risk of postoperative hypoxemia, pulse oximeters and capnography are not always routinely used in practice to monitor patients during transfer from the operating room to the recovery room [81, 82].

The risk of postoperative hypoxemia is increased if the patient is breathing room air during transfer to the recovery room despite preoxygenation and an acceptable minute ventilation, if there is an excessive A-a DO2 [79]. A study by Maity et al. demonstrated that none of the patients recently extubated who received supplemental oxygen during transport developed hypoxemia, while hypoxemia was identified in approximately 30% of patients who were breathing room air during transport [83]. Furthermore, a study by Tyler et al. showed that all patients who developed hypoxemia during transfer with room air returned to normoxia when supplemental oxygen was supplied. The study concluded that supplemental oxygen during transfer may have prevented the incidence of hypoxemia [79]. It is recommended that oxygen supplementation should be given to all patients who have undergone general anesthesia during the transfer from the operating room to the recovery room [18, 80], as well as monitoring by pulse oximeter to detect and treat hypoxemia as soon as possible [1, 83].

## 7. Conclusion

Smooth extubation and successful extubation are often mistakenly considered to be equivalent events. In clinical practice, the goals and advantages of smooth extubation exceed the minimum requirement of maintenance of respiratory function. In this review, we have proposed five factors that should be included in the definition and practice of smooth extubation. They include limited respiratory tract irritation, avoidance of significant hemodynamic shifts, avoidance of iatrogenic injury, maintaining airway patency and physiologic oxygenation/ventilation, and patient comfort.

These factors have previously been described separately and independently, primarily in the context of safety of extubation in general, and anesthesia-related closed claims registries [4, 5]. However, we propose a unifying concept: to include all these factors as a singular definition in order to provide clarity on the concept of smooth extubation. Further research is necessary to establish the utility of using this definition in clinical practice. We believe, however, that adding standardization to the conceptual framework and language of the technique of smooth extubation will help facilitate further research and provide guidance in clinical practice while improving the techniques of extubation and promulgating education in their application.

In our literature review, we have identified three techniques to facilitate smooth extubation: exchanging the endotracheal tube for an LMA prior to extubation, limiting unnecessary stimulation on emergence, such as in the “no-touch technique” described, and deep extubation. Deep extubation in particular may be underutilized in clinical practice due to unconfirmed concerns regarding laryngospasm and aspiration. Additional techniques to mitigate perioperative and postoperative patient harm include use of a bite block, preoxygenation prior to extubation, and use of supplemental oxygen and patient monitoring with pulse oximetry and/or capnography during patient transfer to the recovery room.

In addition, various pharmacologic interventions can be implemented to facilitate the utilization of these techniques. There is clinical evidence to support the use of IV and intracuff lidocaine to attenuate hemodynamic shifts, sore throat, and excessive bucking and coughing during extubation. The use of IV remifentanil and dexmedetomidine has also shown promise to facilitate smooth extubation. Dexmedetomidine may be more effective than remifentanil at preventing postoperative nausea and vomiting. However, it should be noted that dexmedetomidine may result in a slower emergence.

Further research is needed to elucidate the exact clinical circumstances in which these techniques may and should be safely utilized. It is our hope that additional investigations into the application of these pharmacologic and nonpharmacologic techniques with an understanding of the smooth extubation paradigm will improve patient outcomes and reduce the morbidity associated with perioperative extubation.

## Figures and Tables

**Table 1:** Adverse events associated with extubation.

Type of event	Examples
Airway irritation	Coughing
	Bucking on endotracheal tube
	Laryngospasm

Hemodynamic perturbations	Arrhythmia
	Tachycardia/bradycardia
	Hypo/hypertension
	Myocardial ischemia

Airway/oropharyngeal injuries	Larynx including glottis
	Laryngeal nerves
	Oropharynx (tongue, uvula)
	Dentition

Compromised ventilation	Respiratory depression
	Airway obstruction
	Airway edema
	Aspiration
	Pulmonary edema
	Residual neuromuscular blockade

Patient distress	Sore throat
	Hoarse voice
	Unexpected awareness
	Posttraumatic stress disorder

Surgical disruption	Bleeding
	Wound dehiscence
	Flap disruption

**Table 2:** Comparison of pharmacologic adjuncts used in smooth emergence.

Techniques	Intracuff lidocaine	IV lidocaine	IV dexmedetomidine	IV remifentanil	IV calcium channel blockers	Topical benzydamine hydrochloride	Topical methylprednisolone
Proposed advantages	Reduction of postoperative cough, sore throat [53, 54], and dysphonia [55].	Attenuate increases in heart rate and mean arterial pressure [13, 34, 35] and postoperative cough and sore throat [56].	Reduction of postoperative cough [15, 44], nausea, and vomiting [45]. Increased respiratory rate [45]. Decreased agitation and need for rescue analgesic [45, 47].	Attenuate increases in heart rate and mean arterial pressure [36]. Reduction of postoperative cough [36, 44].	Attenuate increases in heart rate and mean arterial pressure [57, 58].	Decreased postoperative sore throat [59, 60].	Decreased postoperative sore throat and cough [61].

Proposed disadvantages	Unknown systemic absorption.	Cardiac arrhythmia local anesthetic toxicity (LAST).	Increased sedation, bradycardia, hypotension [15], time to emergence [47]. Expensive.	Increased postoperative nausea and vomiting. Added expense.	Hypotension. Heart block. Bradycardia.	Burning sensation and possible vomiting when applied [60].	Limited evidence. Systemic steroid absorption. Steroid effects.

**Table 3:** Adjunct maneuvers to maintaining successful extubation.

Maneuver	Description
Preoxygenation with 100% FiO2 [13, 18, 48, 70–72]	Maximizing FRC content of oxygen
Bite block placement before extubation [7, 73–77]	Minimizing endotracheal tube obstruction and oral, lingual, and dental injury
Patient monitoring during transfer to recovery [18, 75, 78–83]	Capnography and/or pulse oximetry during transfer
Supplemental oxygen during transfer to recovery [18, 80, 81]	Preventing hypoxemia during transfer
